# Stearoyl-CoA desaturase 1 and paracrine diffusible signals have a major role in the promotion of breast cancer cell migration induced by cancer-associated fibroblasts

**DOI:** 10.1038/bjc.2015.135

**Published:** 2015-04-16

**Authors:** C Angelucci, G Maulucci, A Colabianchi, F Iacopino, A D'Alessio, A Maiorana, V Palmieri, M Papi, M De Spirito, A Di Leone, R Masetti, G Sica

**Affiliations:** 1 Institute of Histology and Embryology, Faculty of Medicine ‘A. Gemelli', Catholic University of the Sacred Heart, Rome, Italy; 2 Institute of Physics, Faculty of Medicine ‘A. Gemelli', Catholic University of the Sacred Heart, Rome, Italy; 3 Department of Surgery, Breast Unit, Faculty of Medicine ‘A. Gemelli', Catholic University of the Sacred Heart, Rome, Italy

**Keywords:** breast cancer cells, fibroblasts, SCD1, SREBP1, cell migration

## Abstract

**Background:**

Despite the recognised contribution of the stroma to breast cancer development and progression, the effective targeting of the tumor microenvironment remains a challenge to be addressed. We previously reported that normal fibroblasts (NFs) and, notably, breast cancer-associated fibroblasts (CAFs) induced epithelial-to-mesenchymal transition and increases in cell membrane fluidity and migration in well- (MCF-7) and poorly-differentiated (MDA-MB-231) breast cancer cells. This study was designed to better define the role played, especially by CAFs, in promoting breast tumor cell migration.

**Methods:**

Fibroblast/breast cancer cell co-cultures were set up to investigate the influence of NFs and CAFs on gene and protein expression of Stearoyl-CoA desaturase 1 (SCD1), the main enzyme regulating membrane fluidity, as well as on the protein level and activity of its transcription factor, the sterol regulatory element-binding protein 1 (SREBP1), in MCF-7 and MDA-MB-231 cells. To assess the role of SREBP1 in the regulation of SCD1 expression, the desaturase levels were also determined in tumor cells treated with an SREBP1 inhibitor. Migration was evaluated by wound-healing assay in SCD1-inhibited (by small-interfering RNA (siRNA) or pharmacologically) cancer cells and the effect of CAF-conditioned medium was also assessed. To define the role of stroma-derived signals in cancer cell migration speed, cell-tracking analysis was performed in the presence of neutralising antibodies to hepatocyte growth factor, transforming growth factor-*β* or basic fibroblast growth factor.

**Results:**

A two to three fold increase in SCD1 mRNA and protein expression has been induced, particularly by CAFs, in the two cancer cell lines that appear to be dependent on SREBP1 activity in MCF-7 but not in MDA-MB-231 cells. Both siRNA-mediated and pharmacological inhibition of SCD1 impaired tumor cells migration, also when promoted by CAF-released soluble factors. Fibroblast-triggered increase in cancer cell migration speed was markedly reduced or abolished by neutralising the above growth factors.

**Conclusion:**

These results provide further insights in understanding the role of CAFs in promoting tumor cell migration, which may help to design new stroma-based therapeutic strategies.

Over the last decade, research on breast cancer, as well as on other solid tumours, has progressively focused on the elucidation of the intricate web of intercellular interactions occurring between the transformed epithelium and the tumor stroma, composed of mesenchyme-derived cells such as fibroblasts, myofibroblasts, pericytes, endothelial and inflammatory cells. A growing body of experimental evidence indicates that changes taking place in the stromal environment surrounding malignant cells, also called ‘reactive stroma', may have strong functional implications for breast tumor development and progression (Polyak and Kalluri, 2010; Tarin, 2013). These stromal alterations concern gene expression, extracellular matrix deposition/remodelling and secretion of soluble factors, among others (Lebret *et al*, 2007; Polyak and Kalluri, 2010; Tarin, 2013; Gialeli *et al*, 2014). In turn, transformed epithelial cells undergo ‘epithelial-to-mesenchymal transition' (EMT), a reversible switch from a polarised, adherent phenotype to a more motile and invasive one, mainly driven by integrated signalling from the heterogeneous population of ‘cancer-associated fibroblasts' (CAFs) which represent the major cell type of the reactive tumor stroma (Lebret *et al*, 2007; Tarin, 2013). On the basis of gene-expression profile and phenotype, CAFs can be distinguished from resting normal fibroblasts. They release growth factors (e.g., SDF-1*α*, VEGF, HGF, EGF, FGFs, TGF-*β*, PDGF), pro-tumorigenic inflammatory signals and matrix metalloproteases promoting angiogenesis, tumor cell proliferation and invasiveness (Lebret *et al*, 2007; Liao *et al*, 2009; Ostman and Augsten, 2009; Sadlonova *et al*, 2009; Erez *et al*, 2013; Gialeli *et al*, 2014). Tumorigenicity of CAFs derived from breast cancers has been widely demonstrated in animal models (Camps *et al*, 1990; Kuperwasser *et al*, 2004; Orimo *et al*, 2005). Induction of mammary cancers has been reported in mice orthotopically grafted with TGF-*β*- and/or HGF-transfected fibroblasts, co-injected with apparently normal epithelial cells, highlighting the critical role of heterotypic interactions in the development of human breast malignancies (Kuperwasser *et al*, 2004).

Among the various cancer cell signatures, the improvement of the invasive/metastatic tumor cell ability might be considered one of the major outcome of this aberrant dialogue with CAFs (Hu *et al*, 2008; Wang *et al*, 2013). In various human malignancies this increased migratory skill of tumor cells, as well as poor prognosis, significantly correlates with alterations in the motional freedom of lipids and protein molecules in the plasma membrane. In particular, malignant cell membranes have been found to generally exhibit higher fluidity than that of their healthy counterparts (Nakazawa and Iwaizumi, 1989; Taraboletti *et al*, 1989; Zeisig *et al*, 2007).

We have recently shown by co-cultures of mammary cancer cells and fibroblasts their reciprocal influence on various adhesiveness and/or invasiveness features (Angelucci *et al*, 2012). Most notably, an overall pro-tumoral/-invasive effect of CAFs on both well- and poorly-differentiated breast cancer cells has been demonstrated by the induction of EMT and by the increase in membrane fluidity, as well as in migration speed and directness (Angelucci *et al*, 2012).

Saturated fatty acids (SFAs) and monounsaturated fatty acids (MUFAs) are essential elements of membrane phospholipids, regulators of the membrane fluidity and crucial cell signalling mediators controlling various cellular activities (Faergeman and Knudsen, 1997; Tang *et al*, 2002). Alterations in the balance of SFAs and MUFAs can therefore influence a wide variety of cell functions. Stearoyl-CoA desaturase 1 (SCD1), an endoplasmic reticulum-resident Δ9 deasaturase, is one of the main enzyme regulating the fatty acid membrane composition. It catalyses the endogenous biosynthesis of MUFAs from *de novo* synthesised or dietary SFAs and has been recently raised to the role of key regulator of cell growth, programmed cell death and carcinogenesis (Igal, 2011). Abnormally high levels of SCD1 have been reported in human cancers, carcinogen-induced tumours and virus-transformed cells, where the resulting increase in MUFA membrane content has been shown to match with an enhanced membrane fluidity (Li *et al*, 1994; Thai *et al*, 2001; Scaglia *et al*, 2005). In breast cancer cells, pharmacological inactivation or genetic knockdown of SCD1 impairs cancer cell growth and inhibits glucose-mediated lipogenesis (Scaglia *et al*, 2009; Luyimbazi *et al*, 2010). A more loosely packed state of cancer cell membranes has also been reported to correlate with increased migratory capacity (Nakazawa and Iwaizumi, 1989; Taraboletti *et al*, 1989). This latter activity is influenced by paracrine signals too. In particular, the crucial role of CAF-released growth factors such as hepatocyte growth factor/scatter factor (HGF), transforming growth factor-*β* (TGF-*β*) or basic fibroblast growth factor (bFGF) in promoting invasive behaviour of breast tumor cells has been demonstrated (Giulianelli *et al*, 2008; Jedeszko *et al*, 2009; Stuelten *et al*, 2010; Cao *et al*, 2013). This effect well illustrates the dialogue existing between epithelial cancer cells and CAFs in breast tumor milieu. Indeed, the fibroblast contribution to tumor progression through paracrine signalling is, in turn, attributable to the cancer cell ability in altering fibroblast nature, realizing a sort of genome reprogramming of these stromal cells that are ‘instructed' to secrete pro-tumorigenic soluble factors (Stuelten *et al*, 2010; Tyan *et al*, 2011).

The present study was designed to gain further insight into the molecular mechanisms responsible for the fibroblast-elicited alterations of cancer cell membrane fluidity and ability to migrate that we previously reported (Angelucci *et al*, 2012).

With this aim, we co-cultured poorly invasive/low metastasising or highly invasive and metastatic breast cancer cells (MCF-7 and MDA-MB-231, respectively) with fibroblasts isolated from mammary healthy skin (normal fibroblasts (NFs)) or from breast tumor stroma (CAFs). In accordance with the formerly demonstrated enhancement in cancer cell membrane fluidity induced by NFs or CAFs (Angelucci *et al*, 2012), here, we report the ability of fibroblasts to upregulate SCD1 expression. In low invasive tumor cells, this latter effect was mediated by the CAF-triggered induction of the SCD1 transcription factor, sterol regulatory element-binding protein 1 (SREBP1). To assess the alleged involvement of SCD1 in the fibroblast-triggered improvement of cancer cell migration (Angelucci *et al*, 2012), both siRNA knockdown and pharmacologic inhibition of SCD1 was performed in cancer cells with a resulted impairment of their migratory ability. Finally, evaluation of cancer cell migration speed in tumor cell/fibroblast co-cultures in the presence of neutralising antibodies to HGF, TGF-*β* or bFGF, provides evidence of the crucial contribution of these CAF-derived diffusible signals to the CAF promotion of cancer cell motility that we have previously shown (Angelucci *et al*, 2012). In support of this latter data, as well as the crucial role of SCD1 in tumor cell migration, we found that the increase in cancer cell motility induced by the treatment with CAF-conditioned medium was suppressed as a result of the SCD1 pharmacological inhibition.

## Materials and methods

### Clinical specimens and fibroblast cultures

Primary normal mammary skin fibroblasts (NFs) and CAFs were isolated and immunocytochemically assayed to validate their fibroblastic nature as previously described (Angelucci *et al*, 2012). NFs and CAFs were maintained in Dulbecco's Modified Eagle's Medium (DMEM; Euroclone, Milan, Italy) supplemented with 10% foetal bovine serum (FBS; Life Technologies, Carlsbad, CA, USA), antibiotics (100 IU ml^−1^ penicillin, 100 *μ*g ml^−1^ streptomycin, Euroclone), 2 mM glutamine (Euroclone) and 1 mM sodium pyruvate (Euroclone). Cells were incubated at 37 °C in a humidified atmosphere of 5% CO_2_-95% air.

Co-culture experiments were carried out with fibroblasts of passage 2–3 to avoid cell-aging effects.

### Breast cancer cell lines

The estrogen receptor (ER)-positive, poorly invasive and low metastasising human breast carcinoma cell line MCF-7 and the ER-negative, highly invasive and metastatic human breast carcinoma cell line MDA-MB-231 were obtained from American Type Culture Collection (Manassas, VA, USA). MCF-7 and MDA-MB-231 cells were maintained in DMEM (Euroclone) supplemented with 10% FBS (Life Technologies, Paisley, Scotland), antibiotics (100 IU ml^−1^ penicillin, 100 *μ*g ml^−1^ streptomycin, Euroclone) and 2 mM glutamine (Euroclone). Cells were incubated at 37 °C in a humidified atmosphere of 5% CO_2_-95% air.

### Co-cultures

For the evaluation of SCD1 expression, SREBP1 activity/expression and cell migration speed, co-cultures of fibroblasts and tumor epithelial cells were performed. Briefly, subconfluent fibroblast and breast cancer cell cultures were trypsinized and ∼5 × 10^3^ per cm^2^ cells of each cell type were plated together in their standard medium in 100 mm Petri dishes (for the evaluation of SCD1 expression or SREBP1 activity and expression) or in 35 mm glass-bottom Petri dishes (ibidi GmbH, Martinsried, Germany) for cell-tracking analysis of cell migration speed. Medium was renewed after 3 days and all the above analyses were carried out after 6 days, as described below. MCF-7 and MDA-MB-231 homotypic cultures were used as controls.

### Separation of breast cancer cells from the co-cultures

Cells of 6-day monolayer cultures and co-cultures were collected to investigate the influence of NFs and CAFs on SCD1 mRNA and protein level, as well as on SREBP1 protein expression and DNA binding activity in breast cancer cells. Cells in mixed cultures were separated by magnetic cell sorting (EasySep Magnet, StemCell Technologies, Vancouver, BC, Canada) using the Human ‘Do-It-Yourself' Positive Selection Kit (StemCell Technologies) with an anti-fibroblast antibody (anti-human CD90, Thy-1, clone 5E10, StemCell Technologies), according to the manufacturer's instructions. An aliquot of the eluted tumor cell fraction of the co-cultures were analysed by immunoblotting for the expression of the chemokine stromal-derived factor 1 (SDF-1), a marker of cells of mesenchymal origin, in order to determine possible fibroblastic contamination before carrying out quantitative real-time PCR (qRT-PCR) and Western blotting analyses (Supplementary Figure S1).

### qRT-PCR

Because the levels of mammalian SCD1 appear to be principally determined by its rate of transcription (Ntambi *et al*, 2004), we investigated the effect produced by fibroblasts on the desaturase mRNA level in breast cancer cells by qRT-PCR.

Briefly, total RNA was extracted from MCF-7 or MDA-MB-231 cells cultured alone or co-cultured with fibroblasts (NFs or CAFs) for 6 days with the RNAqueous-4PCR kit (Ambion, Austin, TX, USA), according to the manufacturer's instructions. In all 2 *μ*g of RNA was converted into single-stranded DNA by a standard 50-*μ*l RT reaction with the TaqMan RT Reagent (Life Technologies). The cDNA generated from the reverse transcription reactions was amplified by PCR with the TaqMan Gene-expression Assays (Life Technologies) in a total volume of 25 *μ*l. The reactions were incubated at 50 °C for 2 min, 95 °C for 20 s, followed by 40 cycles of 95 °C for 1 s and 60 °C for 20 s, on a StepOne Real-Time PCR Systems (Life Technologies). The primers used were as follows: 5′-CTC CAC TGC TGG ACA TGA GA-3′ (forward), 5′-AAT GAG TGAAGG GGC ACA AC-3′ (reverse); *β*-actin: 5′-ATC TGG CAC CAC ACC TTC-3′ (forward) and 5′-AGC CAG GTC CAG ACG CA-3′ (reverse). The level of SCD1 mRNA was expressed as relative fold change *vs* the *β*-actin mRNA. The relative fold change in mRNA expression was calculated using the 2^−ΔΔCt^ method, where the average of ΔCt values for SCD1 amplicon was normalised to that of *β*-actin, compared with control (MCF-7 or MDA-MB-231 cells cultured alone). Reactions were performed in triplicate.

### Western blot analysis

For SDF-1 and SCD1 analysis, total protein extracts were obtained by lysing tumor cells in RIPA buffer (50 mM Tris-HCl (pH 7.7), 150 mM NaCl, 1% Triton X-100, 1% sodium deoxycholate, 0.1% SDS) freshly supplemented with phosphatase and protease inhibitors (100 *μ*M Na_3_VO_4_, 0.3 mM phenylmethylsulfonylfluoride, 50 *μ*g ml^−1^ leupeptin and 20 *μ*g ml^−1^ aprotinin). To evaluate the expression level of the nuclear mature form of SREBP1, nuclear proteins were extracted using NE-PER Nuclear and Cytoplasmic Extraction Reagents (Thermo Fisher Scientific, Waltham, MA, USA). In all 30 *μ*g of total or nuclear proteins were resolved on a 10% SDS-PAGE and transferred onto Immobilon P membrane (Millipore, Bedford, MA, USA) which was incubated with anti-SDF-1 antibody (Abcam, Cambridge, UK, 1 : 1000), anti-SCD1 antibody (clone M38, Cell Signaling Technology, Danvers, MA, USA, 1 : 2000), or anti-SREBP1 antibody (clone C20, Santa Cruz Biotechnology, Santa Cruz, CA, 1 : 200). The blot was then overlaid with a HRP-labelled secondary antibody (Vector Laboratories, Burlingame, CA, USA; 1 : 3000 dilution). The protein bands were detected using an enhanced chemiluminescence system (ECL, GE Healthcare, Piscataway, NJ, USA) and visualised on Hyperfilm ECL (GE Healthcare). As internal loading control, antibodies to *β*-actin (clone AC15, Sigma-Aldrich, St. Louis, MO, USA, 1 : 10000) and Lamin A/C (clone 14, BD Transduction Laboratories, 1 : 1000) were used for cytosolic (SDF-1 and SCD1) and nuclear (mature active form of SREBP1) targets proteins, respectively. Band intensities were measured by densitometry (Chemi Doc Documentation System/Quantity One quantitation software, Bio-Rad Laboratories Inc, Hercules, CA, USA). Variations in SCD1 or SREBP1 protein levels were expressed as fold change compared with control (MCF-7 or MDA-MB-231 cells cultured alone) after normalisation to *β*-actin or Lamin A/C expression.

### SREBP1 transcription factor activity

To evaluate the influence of NFs and CAFs on the transcription of SCD1 gene in tumor cells, the ability of the mature form of SREBP1 to bind to the SREBP response elements (SREs) on the SCD1 promoter was assayed in MCF-7 or MDA-MB-231 cells cultured alone or co-cultured with fibroblasts for 6 days using an enzyme-linked immunosorbent assay (ELISA; human SREBP1 Transcription Factor Assay Kit, Cayman Chemical, Ann Arbor, MI, USA), according to the manufacturer's instructions. Cancer cell nuclear proteins were extracted using NE-PER Nuclear and Cytoplasmic Extraction Reagents (Thermo Fisher Scientific).

### Small-interfering RNA

The silencing of SCD1 gene was achieved by using small-interfering RNA (siRNA) duplex oligonucleotides against the coding sequence of human SCD1 cDNA that were synthesised and purchased by Integrated DNA Technologies (Coralville, IA, USA). We selected two target sequences as follows: 5′-AAU GAU CAG AAA GAG CCG UAG-3′ (sense_SCD1_1) and 5′AAA UCG UCU CCA ACU UAU CUC-3′ (sense_SCD1_2). Transfection of 60 pmol siRNA in MCF-7 and MDA-MB-231 cells was carried out by using Oligofectamine Transfection Reagent (0.3%, v/v; Life Technologies), according to the manufacturer's instructions. Fresh medium was added 5 h after transfection and cells were further incubated for 72 h. Commercial non-targeting control siRNA NC1 (Integrated DNA Technologies) served as experimental control.

The efficiency of SCD1 downregulation by siRNA was demonstrated by Western blot analysis (Supplementary Figure S2).

### Small-molecule inhibitor treatments

The small-molecule inhibitor of SCD1 (4-(2-chlorophenoxy)-*N*-(3-(methylcarbamoyl)-phenyl)piperidine-1-carboxamide; A939572, Biofine International, Vancouver, BC, Canada) was dissolved in DMSO and used at a concentration of 1 *μ*M to inhibit SCD1 activity in MCF-7 or MDA-MB-231 cells. Briefly, in order to evaluate the effect of SCD1 on tumor cell motility (see ‘Wound-Healing Assay' below) cells were seeded in sixwell plates and grown to subconfluence in their standard medium. They were incubated with 1 *μ*M A939572 or with vehicle (0.01% DMSO, control cells) in serum-free DMEM and the wound closure was evaluated after 24 and 48 h.

In order to evaluate the possible involvement of SCD1 on CAF-promoted tumor cell migration, in a further series of experiments the scratched tumor cell cultures were exposed to the CAF-conditioned medium (CAF-CM), with or without the addition of the SCD1 inhibitor (1 *μ*M), and cell migration evaluated after 24 and 48 h.

To assess the contribution of SREBP1 to the effect induced by CAF-released soluble factors on tumor cell SCD1 expression, the desaturase protein levels were evaluated in MCF-7 and MDA-MB-231 cells exposed to the CAF-CM, with or without the addition of Fatostatin, a small-molecule inhibitor of SREBP activation (Sigma-Aldrich). Briefly, cells were treated for 6 days with 0.2% (v/v) DMSO (vehicle), CAF-CM with 0.2% (v/v) DMSO or 20 *μ*M Fatostatin. SCD1 expression was then determined by Western blotting, as described above.

### Wound-healing assay

In order to verify the effect of the genetic knockdown or pharmacological inhibition of SCD1 on tumor cell motility, a wound-healing assay was performed on MCF-7 and MDA-MB-231 cells transfected with siRNA or treated with the SCD1 inhibitor A939572 (in a series of experiments this latter treatment was also performed on cells exposed to CAF-CM, as described in the previous section). Cells were seeded in sixwell plates and grown to subconfluence. Cell proliferation was blocked by a 2 h pretreatment with mitomycin C (5 *μ*g ml^−1^) in serum-free medium. A scratch was then made in each well using a 200 *μ*l pipette tip and the wounded monolayers washed twice with PBS to remove cell debris and floating cells. The wounds (5 randomly selected points per wound) were photographed at the beginning (time 0) and then after 24, and 48 h under an inverted microscope with a digital camera and the distance migrated by the cells was measured at the reference points by an image-processing software (ImageJ, open source from National Institutes of Health, Bethesda, MD, USA). Three independent experiments were performed with two sets of culture dishes for each condition.

### Growth factor neutralization and cell-tracking experiments

To study the effect of HGF, TGF-*β* and bFGF neutralization on the fibroblast-induced increase in cancer cell migration speed, anti-HGF, -TGF-*β* and -bFGF antibodies were added (alone or combined) to the media of tumor cell cultures and co-cultures (with NFs or CAFs) and tumor migration speed evaluated by single cell-tracking of living cells and time-lapse confocal microscopy, as previously described (Angelucci *et al*, 2012). Cultures and co-cultures in which tumor cells were labelled with the CellTracker Green CMFDA (Life Technologies) were set up in 35 mm glass-bottom Petri dishes (ibidi GmbH). Cells were allowed to grow to 30%–40% confluence in complete medium. Then the cells were incubated in 0.5% FBS-containing DMEM in either the presence or absence of the selected neutralising antibody (monoclonal mouse anti-human HGF, 30 *μ*g ml^−1^; polyclonal rabbit anti-human TGF-*β*, 50 *μ*g ml^−1^; polyclonal goat anti-human bFGF, 10 *μ*g ml^−1^, R&D Systems, Minneapolis, MN, USA) or control non-immune IgGs (mouse, rabbit or goat nIgG, 50 *μ*g ml^−1^, R&D Systems) over the last 48 h prior to perform cell-tracking. Migration speed of CMFDA-labelled tumor cell was evaluated and analysed by using the ImageJ software plugin ‘Particle Tracker' and'Chemotaxis and migration tool'as previously described (Angelucci *et al*, 2012). On average 40 cells were tracked per experiment over a time period of 45 min. The time interval between consecutive frames was 1 min. Once the analysis region was defined by means of the feature point detection and tracking algorithm, each cell was numbered and its (*x(t*_*i*_*), y(t*_*i*_)) position coordinates measured for each time point *t*_*i*_ (*i*=1,…., *N*=45). The instantaneous migration velocity and the mean migration velocity <*v*> for each cell was calculated as 
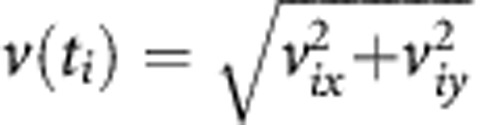
 where *v*_*ix*_ and *v*_*iy*_ were calculated according to the expression:


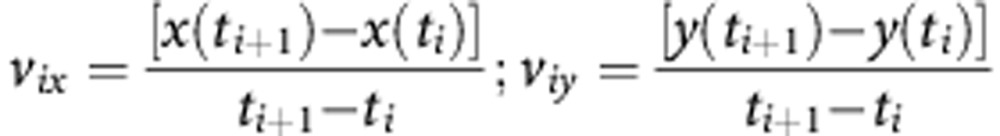


Where (*x(t*_*i+1*_*), y(t*_*i+1*_), (*x(t*_*i*_*), y(t*_*i*_))) are the positions of each cell at the time *t*_*i+1*_ and *t*_*i*_. The mean migration velocity <*v*> was calculated according to the expression:


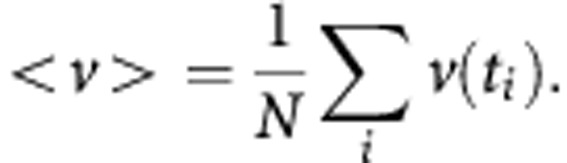


### Statistical analysis

At least three independent experiments were performed for each protocol. The two-tailed Student's *t*-test was used to compare means of two different groups. The level of significance was set at *P*<0.05.

## Results

### Breast CAFs promote SCD1 gene and protein expression in tumor cells

**Figure 1:**
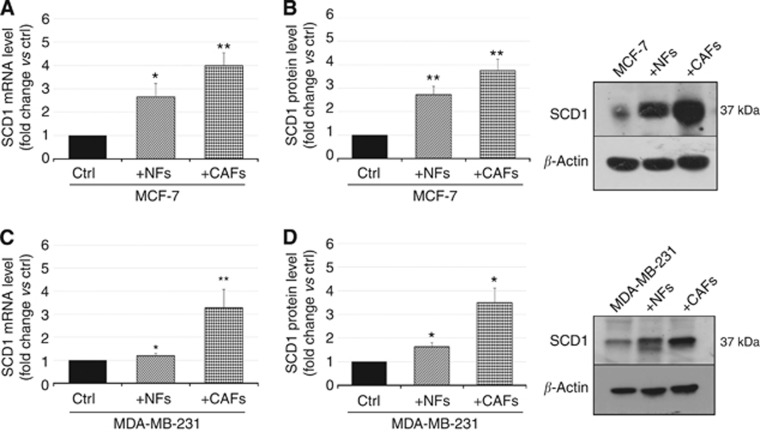
**NFs and CAFs promote SCD1 gene and protein expression in tumor cells.** (**A**, **C**) Analysis of SCD1 mRNA expression by qRT-PCR in the poorly invasive MCF-7 cells (**A**) or in the highly invasive MDA-MB-231 cells (**C**) co-cultured for 6 days with NFs or CAFs revealed a significant NF- or CAF-induced enhancement of SCD1 transcript levels as compared with control (ctrl, MCF-7 or MDA-MB-231 cells cultured alone). (**B**, **D**) Densitometric analysis of SCD1 protein expression in MCF-7 (**B**) or MDA-MB-231 cells (**D**) co-cultured for 6 days with NFs or CAFs (left panels) and representative Western blotting results (right panels). SCD1 protein levels were increased in both the tumor cell lines co-cultured with NFs or CAFs as compared with the ctrl (MCF-7 or MDA-MB-231 cells cultured alone). Variations in SCD1 mRNA and protein levels were expressed as fold change compared with control after normalisation to *β*-actin expression. All experiments were run in triplicate and repeated three times. The data shown are the mean±s.e. *P<0.05 and **P<0.001 *vs* ctrl, Student's *t*-test.

To shed light on the molecular basis of the previously observed increase induced by NFs and CAFs in cancer cell plasma membrane fluidity and migration (Angelucci *et al*, 2012), we co-cultured MCF-7 and MDA-MB-231 cells with NFs or CAFs and investigated the effect of such interaction on SCD1 gene and protein expression in cancer cells. As a result of a 6-day co-culture with NFs or CAFs, a significant induction of SCD1 transcript levels was observed in both MCF-7 and MDA-MB-231 cells (Figure 1A and C). The effect was particularly pronounced in tumor cells co-cultured with CAFs in which a two to threefold enhancement of SCD1 mRNA levels was detected if compared to the control cultures (MCF-7 or MDA-MB-231). Likewise, interactions with fibroblasts induced a statistically significant increase in SCD1 protein expression in tumor cells (Figure 1B and D). As described for gene expression, CAFs showed to be endowed with a higher ability to enhance SCD1 protein levels in cancer cells (by over twofold, compared with control cultures). It is worth pointing out that the efficacy of NFs in promoting SCD1 expression at both mRNA and protein level seemed to undergo a decline in the higher invasive and metastatic breast cancer cells (MDA-MB-231), if compared with MCF-7 cells.

### Breast CAFs differentially affect SREBP1 protein expression and promote SREBP1 DNA binding activity in tumor cells

To identify an upstream target in the control of cancer cell membrane fluidity and migration exerted notably by CAFs, we carried out Western blotting and ELISA and evaluated protein expression and DNA binding activity of the SCD1 transcription factor (SREBP1) in breast cancer cells after 6 days of co-culture with NFs or CAFs.

**Figure 2:**
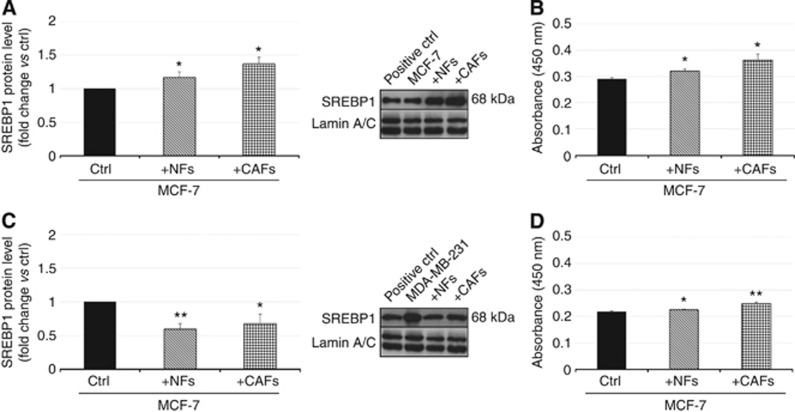
**NFs and CAFs oppositely regulate SREBP1 expression and promote DNA binding activity in MCF-7 and MDA-MB-231 cells.** Nuclear protein extracts were obtained from MCF7 (**A**, **B**) and MDA-MB-231 (**C**, **D**) cells cultured alone (ctrl) or co-cultured with fibroblasts for 6 days and analysed by both Western blotting and human SREBP1 transcription factor assay. Fibroblasts, especially CAFs, induced an increase in both SREBP1 protein level (**A**) and DNA-binding activity (**B**) in MCF-7 cells. In MDA-MB-231 cells, both NFs and CAFs decreased SREPB1 protein levels (**C**) whereas, mainly CAFs, promoted an enhancement of SREBP1 DNA binding activity (**D**). A whole-cell lysate of LNCaP cells was used as a positive control (positive ctrl). Variations in SREBP1 protein levels were expressed as fold change compared with control after normalisation to Lamin A/C expression (**A**, **C**). All experiments were run in triplicate and repeated three times. The data shown are the mean±s.e. *P<0.05 and **P<0.005 *vs* ctrl, Student's *t-*test.

As far as SREBP1 protein expression is concerned, interaction with fibroblasts seemed to produce a divergent effect in the two cancer cell models (Figure 2A and C). In the ER-positive and poorly invasive MCF-7 cells, a statistically significant increase (up to ∼40% compared with control) in SREBP1 protein level was induced, especially as a result of the interaction with CAFs (Figure 2A). Conversely, the co-culture with fibroblasts promoted a downregulation (30–40% compared with control) of SREBP1 in the highly invasive MDA-MB-231 cells (Figure 2C). Consistent with the data from Western blotting (Figure 2A), in MCF-7 cells co-cultured with fibroblasts the DNA binding activity of SREBP1 also underwent a significant increase, mainly as a consequence of co-culture with CAFs (Figure 2B). Despite the reduction in SREPB1 protein level triggered by fibroblasts in MDA-MB-231 cells, an increase rather than a decrease in the transcription factor DNA binding activity was induced in these cells especially by CAFs (Figure 2D).

### SREBP1 pharmacological inhibition impairs CAF-mediated induction of SCD1 expression in poorly invasive but not in highly invasive breast cancer cells

**Figure 3:**
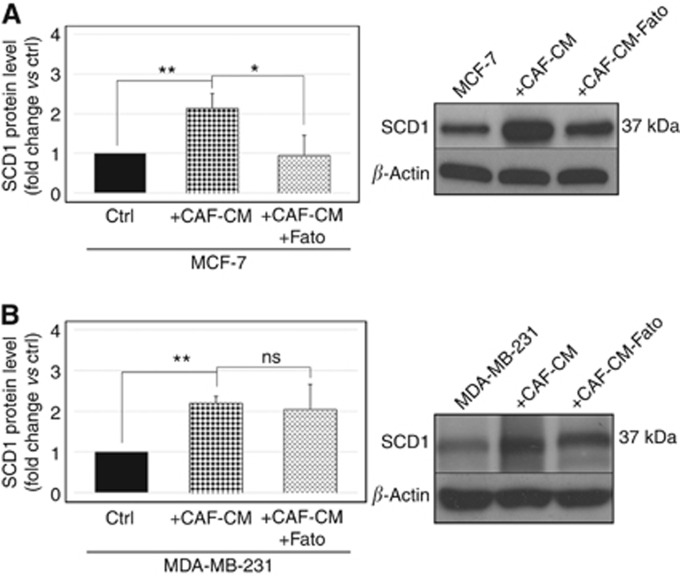
**SREBP1 pharmacological inhibition impairs CAF-mediated induction of SCD1 expression in poorly invasive but not in highly invasive breast cancer cells.** Analysis of SCD1 protein expression by Western blot analysis in the poorly invasive MCF-7 cells (**A**) or in the highly invasive MDA-MB-231 cells (**B**) treated for 6 days with 0.2% (v/v) DMSO (vehicle), CAF-conditioned medium (CAF-CM) with 0.2% (v/v) DMSO or 20 *μ*M Fatostatin (Fato). Densitometric analysis (**A** and **B**, left panels) revealed a significant CAF-induced enhancement of SCD1 levels as compared with control (ctrl, MCF-7 or MDA-MB-231 cells treated with vehicle), which is antagonised by the inhibition of SREBP1 activation by Fatostatin, in MCF-7 (**A**) but not in MDA-MB-231 cells (**B**). Representative Western blots are shown in the right panels. Variations in SCD1 protein levels were expressed as fold change compared with control after normalisation to *β*-actin expression. The data shown are the mean±s.e. of two independent experiments run in triplicate. ns=not significant; *P<0.01 and **P<0.002, Student's *t*-test.

In order to verify the role played by SREBP1 in the CAF-triggered effect on SCD1 expression in tumor cells, the desaturase protein levels were determined by Western blotting in MCF-7 and MDA-MB-231 cells treated with CAF-CM in the presence of 20 *μ*M Fatostatin, an inhibitor of SREPB activation. Exposure of both cell lines to CAF-CM produced a pronounced enhancement of SCD1 levels, comparable to that induced by the co-culture with CAFs (Figure 3). In MCF-7 cells, this effect was completely abolished by the co-incubation with Fatostatin, demonstrating a crucial role for SREBP1 in mediating the CAF-triggered desaturase upregulation (Figure 3A). Conversely, in MDA-MB-231 cells, no significant alteration of the CAF-induced increase in SCD1 expression was observed as a result of Fatostatin treatment (Figure 3B).

### SCD1 genetic inactivation and pharmacological inhibition impair tumor cell migratory ability

**Figure 4:**
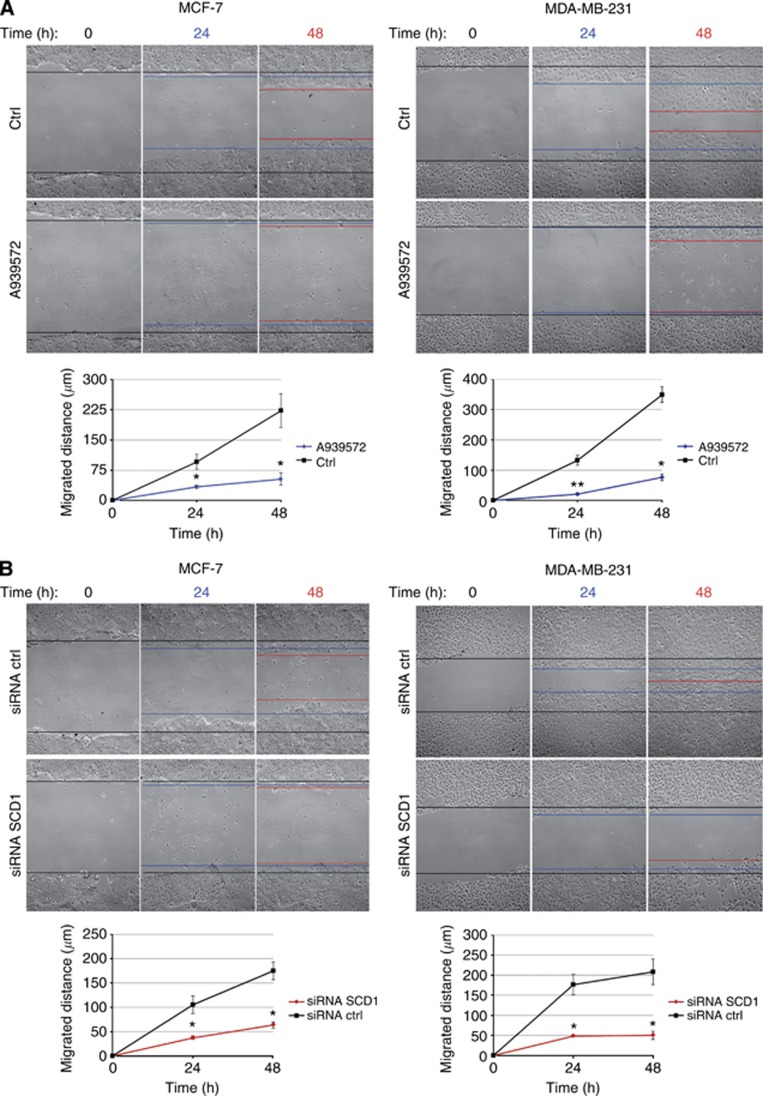
**SCD1 is needed for MCF-7 and MDA-MB-231 cell migration.** (**A**) Pharmacological inactivation of SCD1 with the small-molecule inhibitor A939572 decreased the migratory ability of both MCF-7 and MDA-MB-231 cells in an *in vitro* wound-healing assay. Cells were treated with 1 *μ*M A939572 or with vehicle (control, ctrl) in serum-free DMEM for 24–48 h prior to assay. (**B**) siRNA-mediated knockdown of SCD1 reduced MCF-7 and MDA-MB-231 migration in an *in vitro* wound-healing assay. Cell were transiently transfected for 72 h with 60 pmol of either siRNA ctrl and siRNA SCD1 prior to assay. (**A**, **B**) Cell proliferation was prevented by a 2 h pretreatment with mitomycin C (5 *μ*g ml^−1^). A vertical scratch was made with a pipette tip in the confluent cell monolayers and cell motility assayed at the indicated times (*x*-axis). The gaps were photographed at 0, 24 and 48 h after wounding (original magnification × 100) and the distance migrated was measured in micrometres (*y*-axis) relative to zero time using ImageJ software. All experiments were run in triplicate and repeated three times. The data shown are the mean±s.e. *P<0.05 and **P<0.005 *vs* ctrl (black lines), Student's *t*-test.

The CAF-triggered increase in SCD1 expression is consistent with the CAF-mediated enhancement of cancer cell membrane fluidity and migration that we have previously demonstrated (Angelucci *et al*, 2012). Thus, to assess the possible contribution of SCD1 to breast cancer cell migration, both siRNA-mediated inactivation and pharmacological inhibition of the enzyme were performed in MCF-7 and MDA-MB-231 cells and their migratory activity was evaluated by wound-healing assay. At 24 and 48 h after wounding, a significant reduction in cell migration ability was observed in MCF-7 and MDA-MB-231 cells whose SCD1 expression was either pharmacologically or genetically inhibited (Figure 4A and B, respectively).

Because of the poorly invasive phenotype of MFC-7 cells, at 24 h the impairing effect of both A939572 and siRNA on cell migration was not so striking as at 48 h, when SCD1-depleted cells exhibited a significant reduction in the migrated distance, if compared with control cells (Figure 4A and B, left panels). As expected, in the highly invasive MDA-MB-231 control cells a higher migration rate was observed and a nearly complete or total wound closure was found 48 h after scratching. In these cells, both genetic and pharmacological SCD1 blockade resulted in a dramatic decline of cell migration compared with uninhibited controls (Figure 4A and B, right panels).

**Figure 5:**
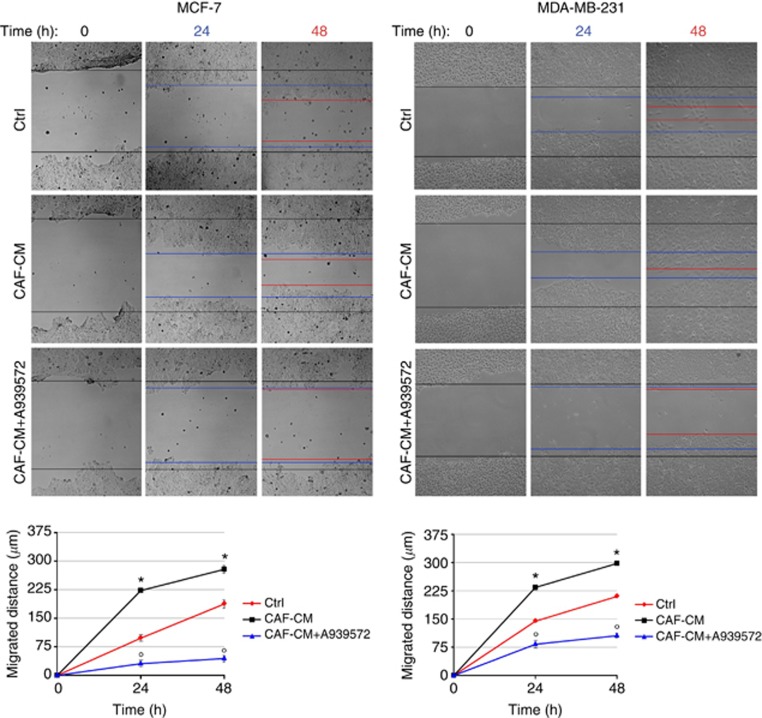
**SCD1 contributes to the promotion of breast cancer cell migration by CAF-derived soluble factors.** Pharmacological inactivation of SCD1 with the small-molecule inhibitor A939572 significantly impaired migration of both MCF-7 and MDA-MB-231 cells which was induced by the exposure to CAF-derived CM (CAF-CM) in an *in vitro* wound-healing assay. Cell proliferation was prevented by a 2 h pretreatment with mitomycin C (5 *μ*g ml^−1^). A vertical scratch was made with a pipette tip in the confluent cell monolayers. Cells were exposed to CAF-CM (with or without 1 *μ*M A939572) or to their standard growth medium (control, ctrl) and the wound closure was evaluated after 24 and 48 h. The gaps were photographed at 0, 24 and 48 h after wounding (original magnification x100) and the distance migrated was measured in micrometres (*y*-axis) relative to zero time using ImageJ software. All experiments were run in triplicate and repeated three times. The data shown are the mean±s.e. *P<0.005 *vs* ctrl (red lines) and °*P*<0.005 *vs* CAF-CM-treated tumor cells (black lines), Student's *t*-test.

In the experiments in which tumor cells were exposed to CAF-CM, a promoting effect of CAF-derived soluble factors on both MCF-7 and MDA-MB-231 cell migration has been found. This effect was completely suppressed by the pharmacological inhibition of SCD1 (Figure 5).

### HGF-, TGF-*β* and bFGF-neutralising antibodies reduce or abolish the migration-promoting effect of CAFs

To test whether secreted endogenous HGF, TGF-*β* and bFGF directly contribute to the fibroblast-triggered enhancement of cancer cell migration speed that we have previously described (Angelucci *et al*, 2012), cell-tracking experiments were performed on tumor cells co-cultured with NFs or CAFs in the presence of antibodies directed against HGF, TGF-*β* or bFGF.

**Figure 6:**
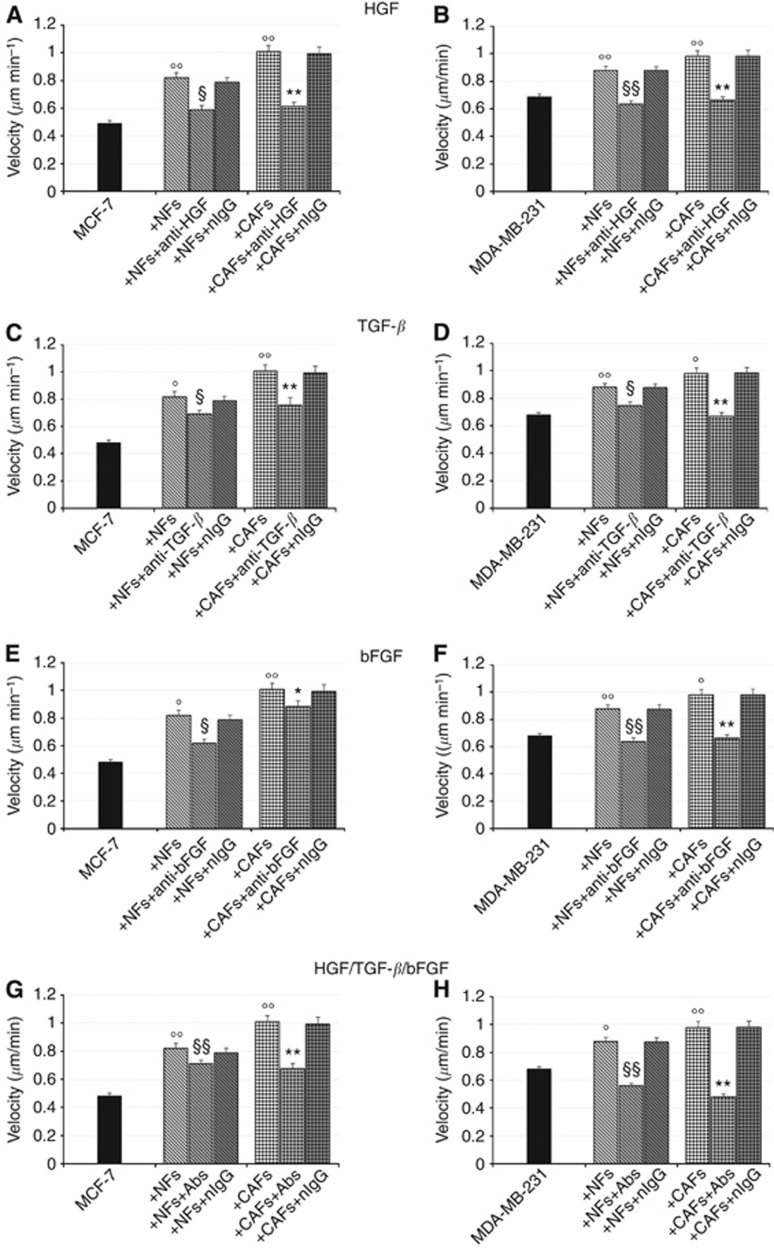
**HGF-, TGF-*β* and bFGF-neutralising antibodies reduce or abolish the NF- and CAF-induced enhancement of cancer cell migration speed.** Cell-tracking experiments were performed on tumor cells co-cultured with NFs or CAFs in the presence of neutralising antibodies to HGF, TGF-*β* or bFGF to investigate the contribution of diffusible signals to the fibroblast promotion of tumor cell migration speed. MCF-7 and MDA-MB-231 cells were cultured for 6 days, alone or in presence of NFs or CAFs, in 35 mm glass-bottom Petri dishes and labelled with the CellTracker Green CMFDA. The cells were incubated in either the presence or the absence of the selected neutralising antibody (anti-HGF, 30 *μ*g ml^−1^; anti-TGF-*β*, 50 *μ*g ml^−1^; anti-bFGF, 10 *μ*g ml^−1^) or control nIgGs (50 *μ*g ml^−1^). Migration speed of CMFDA-labelled tumor cell was evaluated by using the ImageJ software plugin ‘Particle Tracker'. HGF, TGF-*β* and bFGF-neutralising antibodies effectively reduced in MCF-7 (**A**, **C**, **E**) cells or abolished in MDA-MB-231 cells (**B**, **D**, **F**) the fibroblast-induced increases of tumor cell migration speed. When the three antibodies were simultaneously added to the co-cultures neither additive nor synergistic effects were observed (**G**, **H**). All experiments were run in triplicate and repeated three times. The data shown are the mean±s.e. °*P*<0.05 and °°*P*<0.001 *vs* ctrl (MCF-7 or MDA-MB-231 cells cultured alone), ^§^*P*<0.05 and ^§§^*P*<0.001 *vs* MCF-7(+NFs) or MDA-MB-231(+NFs), *P<0.05 and **P<0.001 *vs* MCF-7(+CAFs) or MDA-MB-231(+CAFs) Student's *t*-test.

The addition of the HGF neutralising antibody to the co-culture media proved to be effective in counteracting the fibroblast-elicited increase in tumor cell migration speed (Figure 6A and B). As far as MCF-7 cells are concerned, both the NF- and CAF-triggered migration-promoting effects were significantly reduced by the addition of the anti-HGF antibody (Figure 6A), whereas they were completely abolished in MDA-MB-231/fibroblast co-cultures (Figure 6B).

The effects of TGF-*β* neutralization in MCF-7/ or MDA-MB-231/fibroblast co-cultures were almost superimposable to those described for HGF (Figure 6C and D).

The use of a neutralising antibody to bFGF led to a suppression or a significant reduction of the increase in MCF-7 cell migration speed induced by NFs or CAFs, respectively (Figure 6E). In the highly invasive MDA-MB-231 cells co-cultured with fibroblasts, the migration-promoting effects induced by both NFs and CAFs were completely nullified by the treatment with an anti-bFGF antibody (Figure 6F), as was found for HGF and TGF-*β* neutralization.

Neither additive nor synergistic effects were observed when the three neutralising antibodies were simultaneously added to the co-cultures (Figure 6G and H).

Overall, results obtained in MCF-7 cells suggest that HGF might be the major factor involved in the CAF-induced promotion of tumor cell migration speed, whereas for the highly invasive MDA-MB-231 cells the three growth factors seem to almost equally contribute.

In all the experiments, treatment with nIgG had no effect on fibroblast-induced promotion of cancer cell migration (Figure 6A and H).

Interestingly, none of the neutralising antibodies used seemed to affect the basal migration rate of MCF-7 cells (Supplementary Figure S3A, S3C and S3E), suggesting a critical paracrine contribution by tumor stroma to the observed improvement in cancer cell migratory skill. Conversely, neutralization of each of the three secreted growth factors resulted in a certain reduction of the basal migration speed of MDA-MB-231 cells (Supplementary Figure S3B, S3D and S3F), which, as expected, was higher than that of MCF-7. This finding seems to indicate that also autocrine HGF, TGF-*β* and bFGF signalings might contribute, albeit to a lesser extent, to migration in these highly invasive tumor cells.

## Discussion

In breast cancer research, the critical supporting role of the tumor microenvironment as a source of signalling promoting tumor cell growth and invasiveness has been increasingly recognised (Polyak and Kalluri, 2010; Tarin, 2013).

We have recently reported the reciprocal influence of mammary cancer cells and fibroblasts isolated from mammary skin or tumor stroma on structural and functional aspects of their adhesion and migratory phenotype (Angelucci *et al*, 2012). Notably, CAFs have been demonstrated to induce EMT-related molecular changes, as well as increases in cell membrane fluidity and motility in both well- and poorly-differentiated breast cancer cells (Angelucci *et al*, 2012). Hence, this study was aimed to clarify the role played notably by CAFs in improving breast tumor cell migratory ability through the investigation of the expression/activity of upstream potential effectors, as well as of the influence of paracrine signalling.

In this report, we demonstrated the major contribution of SCD1 and secreted growth factors to the fibroblast-mediated promotion of cancer cell migration we have previously described (Angelucci *et al*, 2012). To the best of our knowledge, this is the first study demonstrating the ability of NFs and CAFs to induce an enhancement of both transcript and protein levels of SCD1. By steadily converting SFA into MUFA, SCD1 can be considered the main enzyme regulating lipid bilayer fluidity. In addition, its key role in cancer development and progression is widely recognised (Enoch *et al*, 1976; Igal, 2011). Although the growing number of studies on lipid metabolism alterations, this issue still represents a quite neglected area of cancer research. Indeed, inhibition of several enzymes involved in fatty acid synthesis, which are expressed at high levels in malignant cells, impairs cell growth and promotes apoptosis (Swinnen *et al*, 2000; Scaglia *et al*, 2005, 2009; Pandey *et al*, 2012; Zaidi *et al*, 2012). This is the case for SCD1, whose overexpression have been described in several tumours and transformed cells (Li *et al*, 1994; Thai *et al*, 2001; Scaglia *et al*, 2009). In breast cancer cells, pharmacological, as well as genetic inactivation of SCD1 inhibits tumor cell proliferation and glucose-mediated lipogenesis (Scaglia *et al*, 2009; Luyimbazi *et al*, 2010).

We have previously reported the CAFs' ability to promote an increase in plasma membrane fluidity of breast tumor cells (Angelucci *et al*, 2012). High SCD1 protein and activity levels, leading to an increase in MUFA membrane content, are acknowledged to be critical features allowing a more fluid membrane organisation to be achieved (Stubbs and Smith, 1984; Scaglia *et al*, 2005). Thus, the CAF-induced upregulation of SCD1 gene and protein expression that we found in either poorly or highly invasive tumor cells appears to match the above data well.

Enhanced membrane fluidity, in turn, has been found to correlate with an increased migratory/invasive activity and malignant potential of tumor cells, as well as with a poor prognosis (Nakazawa and Iwaizumi, 1989; Taraboletti *et al*, 1989; Zeisig *et al*, 2007). To this regard, we have also described the ability of CAFs to significantly improve mammary tumor cell migration speed (Angelucci *et al*, 2012). In virus-transformed human lung fibroblasts, as well as in human lung adenocarcinoma cells, SCD1 knockdown led to a reduction in the anchorage-independent growth, suggesting a critical role for the desaturase in controlling cancer cell invasiveness and metastatic potential (Scaglia and Igal, 2005, 2008). Consistent with these findings, herein, we observed a significantly impaired migratory activity of both MCF-7 and MDA-MB-231 cells tumor cells as a result of either siRNA-mediated silencing or pharmacological inhibition of SCD1. On the basis of the above evidence, we could reasonably assume that the fibroblast-induced upregulation of SCD1 gene and/or protein expression in tumor cells substantially contributes to the previously observed increases in both cancer cell membrane fluidity and migration triggered by CAFs (Angelucci *et al*, 2012).

Given the recognised role of SREBP1 as a powerful activator of SCD1 gene transcription (Horton *et al*, 2003; Ettinger *et al*, 2004), we investigated the effects of NFs and CAFs on its expression and ability to bind to the SREs on the SCD1 promoter in tumor cells. As expected, a significant increase in SREBP1 level and activity was induced, notably by CAFs, in the poorly invasive MCF-7 cells. The crucial involvement of SREBP1 in the induction of SCD1 levels promoted particularly by CAFs in mammary tumor cells is even more clearly demonstrated by the results of SREBP1 pharmacological inhibition which, in MCF-7 cells, led to the disappearance of the SCD1 upregulation promoted by CAF-derived paracrine signalings. A similar concomitant regulation of SCD1 and SREBP1 has also been described in the same cell model by other authors who found that the rapamycin-mediated inhibition of mTOR signalling led to a decrease in SCD1 transcript and protein levels, as well as in SREBP1 expression and DNA binding activity (Luyimbazi *et al*, 2010). Interestingly, in several human normal and neoplastic cell lines, including MDA-MB-468 breast cancer cells, impairment of mTOR pathway by rapamycin was found to inhibit cell motility or invasion (Zhou and Huang, 2011). These findings support the existence of a *fil rouge* linking SREBP1/SCD1-dependent regulation of fatty acids membrane composition of breast tumor cells to their migratory behaviour. Unexpectedly, in the highly aggressive MDA-MB-231 cells, despite a fibroblast-promoted enhancement of SREBP1 DNA binding activity, a sharp reduction in the protein level was induced by both NFs and CAFs. This apparent discrepancy between SREBP1 expression and activity may be consistent with a hyperactivation of the residual protein counterbalancing its scarceness. A dual regulation of the expression of SREBP1 and its downstream effector SCD1 has been previously described in prostate cancer tissue specimens where high and low levels of SREBP1 and SCD1, respectively, have been detected (Ettinger *et al*, 2004; Moore *et al*, 2005). Also, it should be considered that MDA-MB-231 are poorly-differentiated cells, displaying a mesenchymal-like phenotype. If, on the one hand, this feature supports their invasive behaviour, on the other hand, it could lead to gene and protein dysregulation, if compared with well-differentiated breast tumor cells (Neve *et al*, 2006). Thus, in this cell model, also SREBP1 independent mechanisms may partly account for SCD1 upregulation. Indeed, in support of this hypothesis, we found that the inhibition of SREBP1 activation by Fatostatin in MDA-MB-231 cells failed to produce any significant effect on the increase in SCD1 protein levels induced by CAF-secreted factors.

In various human nonmalignant cells SCD1 has been found to be a target of hormones and growth factors, including TGF-*β* and bFGF, which upregulate the desaturase expression (Samuel *et al*, 2002; Demoulin *et al*, 2004). The crucial role played by CAF-derived diffusible signals in controlling cancer cell migration and invasiveness has been also well-established (Bhowmick *et al*, 2004; De Wever *et al*, 2008). Therefore, we investigated the alleged contribution to the fibroblast-mediated increase in cancer cell migration speed of three CAF-released growth factors which are believed to greatly influence tumor spread, such as HGF, TGF-*β* or bFGF (Bhowmick *et al*, 2004; De Wever *et al*, 2008). The antibody-mediated neutralization of these endogenous growth factors in cancer cell/fibroblast co-cultures respectively reduced or abolished the stimulatory effect produced notably by CAFs on MCF-7 and MDA-MB-231 cell migration speed. These findings suggest a relevant impact of the CAF-released signals on the improvement of the tumor cell migratory ability induced by fibroblasts. SCD1 activity seems to have a crucial role in eliciting such effect, as demonstrated by the complete suppression of the increase in tumor cell migration induced by the exposure to the CAF-CM, as a result of the SCD1 pharmacological inhibition. Nevertheless, the lack of a complete abolition of the fibroblast stimulatory effect in the poorly invasive MCF-7 cells provides evidence of a more complex signalling network responsible for CAF-promoted tumor cell migration in welldifferentiated/low aggressive breast cancers. Further, we found that the highly invasive MDA-MB-231 cells are able to produce a certain amount of HGF, TGF-*β* or bFGF, which slightly influenced their migratory speed. Thus, the suppressive effects observed in the presence of neutralising antibodies may be also owing to the inhibition of these autocrine loops. A possible CAF-triggered secretion of HGF, TGF-*β* or bFGF by cancer cells cannot be excluded as a contributory cause of the observed effects. Production of large amounts of the HGF activator (HGFA), stimulating expression of HGF in stromal cells, has been previously demonstrated in highly invasive breast cancer cells such as MDA-MB-231 that also overexpress the HGF receptor c-met. Conversely, the poorly invasive MCF-7 cells displayed low levels of c-met and HGFA (Parr and Jiang, 2006). Ribozyme transgenes targeting of HGF in human fibroblasts or c-met in MDA-MB-231 tumor cells resulted in a reduction of mammary cancer cells *in vitro* invasiveness (Jiang *et al*, 2003). Normal mammary fibroblasts engineered to secrete HGF have been reported to increase invasiveness of c-met expressing human ductal carcinoma *in situ* (DCIS) cells *in vitro* and promote the *in vivo* transition of nude mice DCIS cell xenografts to invasive carcinomas (Jedeszko *et al*, 2009). Likewise, forced overexpression of HGF or TGF-*β* in human mammary fibroblasts can induce human mammary epithelia to develop outgrowths indistinguishable from invasive breast carcinomas (Kuperwasser *et al*, 2004). Consistent with our findings, migration and scattering of the human metastatic breast cancer cells MCF10CA1a has been demonstrated to be dependent on TGF-*β* secretion by normal murine dermal fibroblasts (Stuelten *et al*, 2010).

Compelling evidence has also been provided of the influence of CAF-derived bFGF on mammary cancer hormone-independent growth (Giulianelli *et al*, 2008). In cultured human breast cancer cells, including MDA-MB-231, bFGF has been found to induce migration whereas a correlation between bFGF expression in stromal fibroblasts and high tumor invasiveness has also been described in other cancer types, such as oral squamous-cell carcinoma (Hase *et al*, 2006).

In summary, our study provides additional information to help define the molecular basis of the migration-promoting role of CAFs in breast cancer. In the complex network of epithelium-stroma interaction, the identification of further elements in the CAF-regulated signalling axis controlling mammary cancer cells migration may offer new molecular objectives. Notably, the relevant inhibitory effects produced by SCD1 depletion on mammary cancer cells migration further consolidate the role of this enzyme as one of the convincing future candidate therapeutic target. It is worth mentioning that NFs, used as a ‘normal' control to evaluate the CAFs' impact on cancer cells, triggered overall similar effects as those mediated by CAFs, even though at a lesser extent. This could be ascribable to the probable alterations that these normal cells may go through during their cohabitation in co-culture with neoplastic cells.

## Supplementary Material

Supplementary Figure Legneds
