# Supplementary material for: Stearoyl-CoA desaturase 1 and paracrine diffusible signals have a major role in the promotion of breast cancer cell migration induced by cancer-associated fibroblasts
Source: Br J Cancer. 2015 Apr 16;112(10):1675–86. doi: 10.1038/bjc.2015.135 (PMC4430719; doi:10.1038/bjc.2015.135)
Supplement: Supplementary Figure Legneds [file bjc2015135x1.doc]

**SUPPLEMENTARY FIGURE LEGENDS**

**Supplementary Figure S1**

**Post-sort check of breast cancer cell populations purity**. Purity check of MCF-7 (A) and MDA-MB-231 (B) cells co-cultured with NFs or CAFs and separated by magnetic cell sorting (EasySep™ Magnet, StemCell Technologies) was carried out by analysing an aliquot of the eluted tumor cell fraction by immunoblotting for the expression of SDF-1, a marker of cells of mesenchymal origin, in order to determine possible fibroblast contamination. Thirty µg of total proteins were resolved on a 10% SDS-PAGE and transferred onto Immobilon P membrane (Millipore, Bedford, MA, USA) which was incubated with anti-SDF-1 antibody (Abcam, 1:1000). A whole cell lysate (30 µg) of human fibroblasts obtained from gingival hyperplasia was used as a positive control (positive ctrl). SDF-1 expression levels were normalized to β-actin. Results indicate the absence of a fibroblast contamination in tumor cell samples, as a complete lack of signal both in breast cancer cells cultured alone and in those derived from co-cultures was found.

**Supplementary Figure S2**

**Efficiency of siRNA-mediated SCD1 knockdown**. Sixty pmol of two Small interfering RNA duplex oligonucleotides (SCD_1 and SCD_2), complementary to the coding sequence of human SCD1 cDNA were used to transiently transfect MCF-7 and MDA-MB-231 cells for 72 h. Non-targeting siRNA (control, ctrl) was used as a negative control for evaluating RNA interference off-target effects. The silencing efficiency of both siRNAs was evaluated by Western blot analysis using an anti-SCD1 antibody (clone M38, Cell Signaling Technology). SCD1 protein levels in siRNA-silenced cells were compared to those of ctrl after normalization to β-actin expression.

**Supplementary Figure S3**

**HGF-, TGF-β, and bFGF-neutralizing antibodies do not influence or slightly affect migration speed of cancer cells cultured alone.** Cell tracking experiments were performed on tumor cell cultures in the presence of neutralizing antibodies to HGF, TGF-β or bFGF to investigate the possible contribution of cancer cell-derived diffusible signals to tumor cell migration speed. MCF-7 and MDA-MB-231 cells were cultured for 6 days in 35 mm glass-bottom Petri dishes and labelled with the CellTracker Green CMFDA. The cells were incubated in either the presence or the absence of the selected neutralizing antibody (anti-HGF, 30 µg/ml; anti-TGF-β, 50 µg/ml; anti-bFGF, 10 µg/ml) or control nIgGs (50 µg/ml). Migration speed of CMFDA-labelled tumor cell was evaluated by using the ImageJ software plugin “Particle Tracker”. HGF, TGF-β and bFGF neutralizing antibodies did not affect MCF-7 cell migration speed (A, C, E) which was slightly inhibited in MDA-MB-231 cells (B, D, F).

All experiments were run in triplicate and repeated three times. The data shown are the mean ± SE. *p<0.05 vs MCF-7 or MDA-MB-231 cells, Student’s *t* test.
