# Supplementary figures and images for: Stearoyl-CoA desaturase 1 and paracrine diffusible signals have a major role in the promotion of breast cancer cell migration induced by cancer-associated fibroblasts
Source: Br J Cancer. 2015 Apr 16;112(10):1675–86. doi: 10.1038/bjc.2015.135 (PMC4430719; doi:10.1038/bjc.2015.135)

A

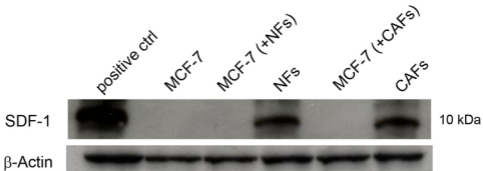

B

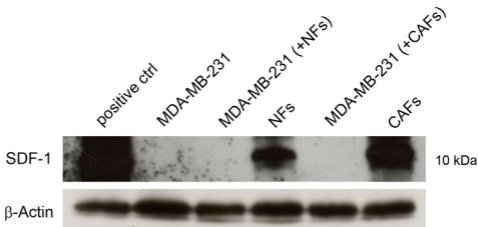

Supplement: Supplementary Figure S1 [file bjc2015135x2.pdf]

## siRNAs

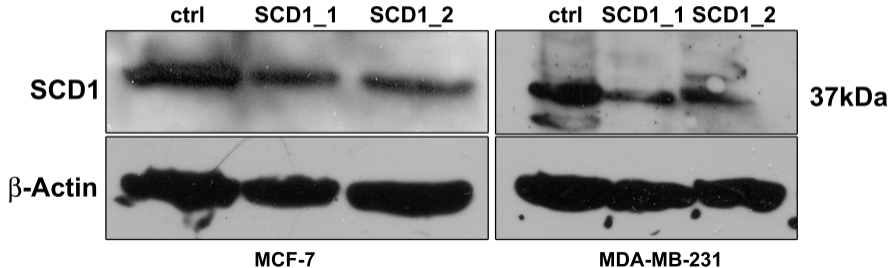

Supplement: Supplementary Figure S3 [file bjc2015135x3.pdf]

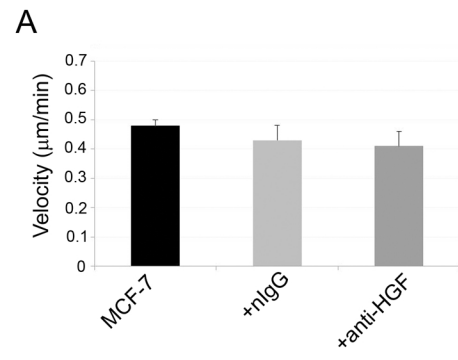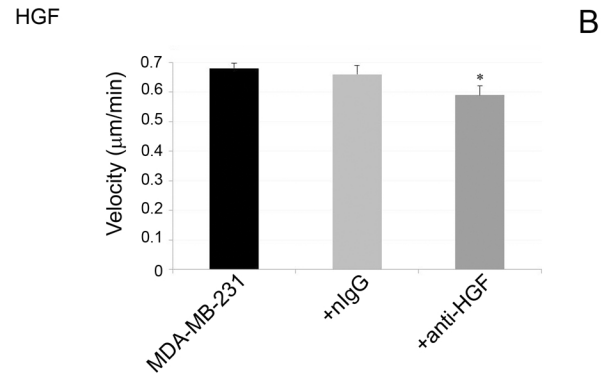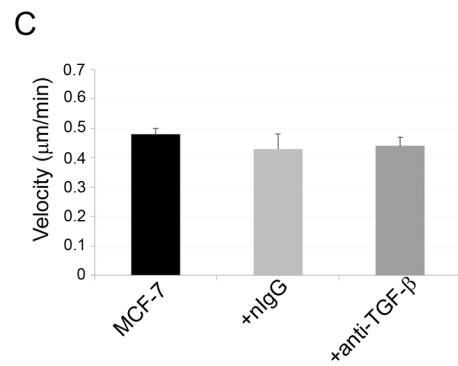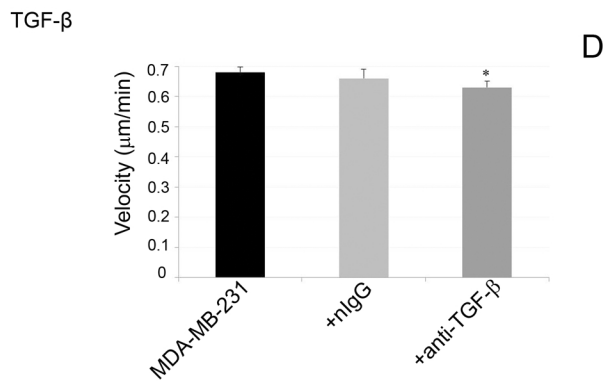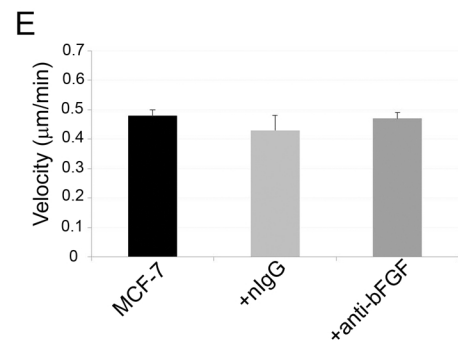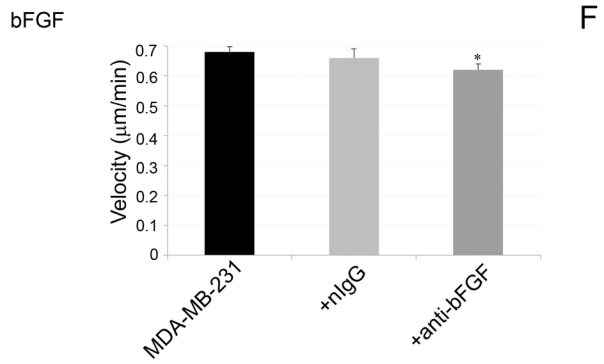

Supplement: Supplementary Figure S3 [file bjc2015135x4.pdf]
